# A complex legacy: USAID, population control, and reproductive justice in Africa

**DOI:** 10.1080/26410397.2026.2633926

**Published:** 2026-02-20

**Authors:** Nimrod Muhumuza, Nana Koomson

**Affiliations:** a Head of Evidence Generation, Afya na Haki, Kampala, Uganda; b Research Officer, Afya na Haki, Kampala, Uganda.

**Keywords:** population control, USAID, Africa, reproductive justice, sexual and reproductive health and rights

## Abstract

USAID's interventions have contributed significantly to improving maternal and child health, preventing HIV, and combating child marriage and gender-based violence. However, the agency's dissolution has highlighted the urgent need to reckon with its population control history. USAID's covert population control initiatives in the Global South were predicated on the flawed premise that burgeoning populations, rather than structural inequalities, were the primary cause of poverty and underdevelopment in those territories. These population policies, advanced by USAID in the early years of its work, resulted in violations of sexual and reproductive rights and were designed to secure Western access to agricultural land and mineral wealth on the African continent. Given this troubling history, this article argues that USAID must formally confront its past through institutional acknowledgement, establishment of comprehensive reparative mechanisms, and prioritisation of indigenous sexual and reproductive health and rights (SRHR) programming alongside domestic resource mobilisation.

## Résumé

Les interventions de l’USAID ont largement contribué à l’amélioration de la santé maternelle et infantile, à la prévention du VIH ainsi qu’à la lutte contre les mariages d’enfants et les violences sexistes. Néanmoins, la dissolution de l’institution a souligné qu’il était urgent pour l’USAID d’assumer son passé en matière de contrôle des naissances. Les initiatives dissimulées de contrôle des naissances menées par l’USAID dans les pays du Sud reposaient sur le postulat erroné selon lequel la croissance démographique, plutôt que les inégalités structurelles, était la principale cause de pauvreté et de sous-développement dans ces territoires. Ces politiques démographiques, mises en œuvre par l’USAID au début de son activité, ont entraîné des violations des droits sexuels et reproductifs, et visaient à garantir aux Occidentaux l’accès aux terres agricoles et aux richesses minières du continent africain. Face à ce passé troublant, l’article avance que l’USAID doit officiellement assumer son histoire par une reconnaissance institutionnelle, l’établissement de mécanismes globaux de réparation et la priorisation des programmes de santé et droits sexuels et reproductifs destiné aux populations autochtones, parallèlement à la mobilisation de ressources nationales.

## Resumen

Las intervenciones de USAID han contribuido de manera significativa a mejorar la salud materno-infantil, prevenir el VIH y combatir el matrimonio infantil y la violencia de género. Sin embargo, la disolución de la agencia ha puesto de relieve la necesidad urgente de enfrentar su historial de control de la población. Las iniciativas encubiertas de USAID para controlar la población en el Sur Global se basaron en la premisa errónea de que las poblaciones en aumento, y no las desigualdades estructurales, eran la principal causa de pobreza y subdesarrollo en esos territorios. Estas políticas de población, promovidas por USAID en los años iniciales de su trabajo, resultaron en violaciones de los derechos sexuales y reproductivos y fueron diseñadas para asegurar el acceso de Occidente a tierras agrícolas y riqueza mineral en el continente africano. En vista de este historial preocupante, este artículo argumenta que USAID debe confrontar su pasado formalmente mediante el reconocimiento institucional, el establecimiento de mecanismos reparadores integrales y la priorización de programas de salud y derechos sexuales y reproductivos (SDSR) de indígenas junto a la movilización de recursos nacionales.

## Introduction

On 10 March 2025, the United States Agency for International Development (USAID) drastically cut 83% of its programmes, with the remaining initiatives absorbed by the State Department on 1 July 2025.^1,2^ USAID's absence will result in an estimated 4.2 million unintended pregnancies and 14 million additional deaths by 2030.^3,4^ In response, leaders, especially those in Africa whose health systems were reliant on USAID funding, were urged to shift from foreign funding to domestic revenue. However, many African nations are burdened with debt, limiting their ability to cover the resulting shortfalls.^5^

USAID was the largest bilateral funder of health and family planning in Africa, in the 2023 fiscal year, managing approximately US$40 billion – less than 1 percent of the United States (US) federal budget.^6^ As of 2023, the Agency allocated over US$600 million to sexual and reproductive health services globally. This supported integrated programmes that combined family planning with maternal and child health, HIV prevention, and efforts to end child marriage and gender-based violence.^7^ USAID's support has shaped the view from practitioners within the sexual and reproductive health space that SRHR is a shared responsibility that requires action from several actors, including civil society organisations, educators, the media, opinion leaders, and governments.

As we reckon with its sudden absence, it is crucial to examine USAID's legacy in its entirety, especially its historical role in shaping reproductive health policies in Africa. Understanding this past not only ensures USAID's institutional accountability but also enables us to identify and prevent the injustices that have long undermined reproductive autonomy across the continent. This reckoning allows us to transform USAID's absence from a moment of financial loss into an opportunity to challenge dependency, confront historical injustices, and reimagine reproductive justice through an African-led and historically conscious lens.

This paper interrogates USAID's coercive population control history in Africa and argues that a progressive SRHR landscape on the continent requires formal acknowledgement of this history and the establishment of meaningful reparative mechanisms by the Agency or the entities that have absorbed its functions. The paper traces the genealogy of USAID's coercive population control agenda, examines how the Agency has simultaneously advanced and undermined reproductive justice, and concludes with concrete recommendations for accountability and African-led SRHR governance.

## Original sin: USAID and population control in Africa

Legacies are complicated, and none more so than USAID's. To speak of SRHR and reproductive justice in Africa requires shedding light on its inconvenient truths and, most importantly, demanding accountability for its oppressive population control history. While its contemporary work has improved access to sexual and reproductive health services for many African women and girls, we cannot mourn the loss of USAID without recognising the “original sin” inherent in its creation and its role within the population control movement in the Global South. Ignoring or whitewashing these narratives without “disinfecting them with sunlight” allows anti-gender and anti-rights actors to manipulate and distort that history and, most importantly, undermine the work of the reproductive justice movement today.^8^ These anti-rights groups highlight and exploit the eugenicist and racist histories of many organisations, including USAID, that are now fighting the good fight for equitable sexual and reproductive health and rights for all.

Organisations such as MSI Reproductive Choices and Planned Parenthood have confronted, and continue to confront, this troubled past, acknowledging their previous involvement in population control and integrating rights and evidence-based mechanisms that uphold participatory and ethical principles in their SRHR initiatives.^9,10^ However, no formal apology or institutional admission of complicity in eugenics or coercive population control policies has been made by USAID. Similarly, there has been no reparative mechanism or public accountability for the harms it caused in specific countries in the Global South, including Africa. The following section traces USAID's propagation of the population movement in Africa.

## Genealogy of USAID's population control in Africa

In the 1960s and 1970s, there was a prevailing belief among proponents of the population control movement in the US that the economic advancement of Global South countries depended on reducing fertility rates.^11^ This view was further justified by the claim that poverty and underdevelopment stemmed from large populations rather than structural issues.^12^ Within this framing, population concerns in the Global South centred on women of reproductive age, who became prime targets of coercive population programmes and policies. Impoverished women and girls were rendered vulnerable by Malthusian-inspired assumptions propagated by Global North policy makers who blamed them for their own poverty and underdevelopment. Family planning among African populations, for example, was promoted by these policy makers as a cure for social ills, including misery in urban and rural ghettos, and rising crime and vagrancy rates across the continent. African women and girls were depicted as sexually promiscuous and ignorant by proponents of Malthusianism, and therefore incapable of using contraceptive methods that required user compliance. Such assumptions provided justification for intrusive and coercive reproductive interventions.^13^

The legislative framework guiding USAID, notably the Foreign Assistance Act of 1961, explicitly included population control among the criteria for aid allocation, emphasising population stabilisation as essential to effective development assistance.^14^ The Act stressed that uncontrolled population growth in the Global South would undermine otherwise successful development efforts in those regions, and encouraged the provision of US assistance aimed at increasing motivation for family planning and reducing population growth rates.^14^

Thus, USAID began its family planning programme in 1965 as an initiative to counter population growth, specifically, in the Global South.^15^ The Agency distributed such vast quantities of contraceptives that it faced difficulties managing its inventory and preventing unused condoms and pills from expiring in storage facilities.^16^ Although population growth was presented by USAID as a critical development, health, and human rights concern, this programmatic impetus did not originate from an overarching commitment to uphold human or sexual and reproductive rights.^15^ As Horn explains, family planning was not viewed as a rights issue for the US and USAID at that time. Instead, there was a concern that increasing populations or “the population problem” could threaten US foreign security and challenge US access to minerals and other natural resources.^17^

Building on this critique, Martin argues that Western powers consciously and systematically pursued policies to control, reduce, and even eliminate Africa's population in order to secure exclusive access to fertile agricultural land and vast mineral wealth.^18^ This racialised logic was particularly evident in South Africa, where Kuumba (1993) notes that the unchecked growth of indigenous African populations was framed as a threat to white power, safety, and profits. By 1983, this translated into the establishment of approximately 21,000 birth control clinics, which targeted African women and offered only contraceptive devices.^13^

These geopolitical and economic imperatives shaped not only the rationale for US involvement but also the methods through which population control policies were implemented. From the 1960s onwards, funding for international population policy activities expanded significantly, yet the United States made considerable efforts to conceal the extent of its involvement in overseas programmes in order to protect its own interests and avoid oversight. USAID, in particular, was instructed to remain out of sight and avoid public scrutiny, often funnelling resources through intermediaries such as the Pathfinder Fund.^19,20^ Through these middlemen, USAID became a central actor in implementing US government family planning initiatives, working with governments and non-governmental organisations across the Global South.

For example, in Senegal, USAID conditioned the provision of aid on the adoption of population control.^21^ Further, in Egypt, USAID required the institutionalisation of population control as a development priority, which was embedded within complex geopolitical and democratisation projects.^22^ In Nigeria, USAID promoted a population policy that integrated family planning into its broader health programme. This initiative, however, was not focused on supporting Nigerians in achieving their desired family size. Instead, it outlined a range of activities that extended beyond what was culturally acceptable or religiously permissible in the Nigerian context. Indeed, the central aim of the programme appeared to be to prevent as many Nigerians as possible from having children.^19^ Notably, USAID officials were instructed to exert maximum leverage and influence to ensure that governments in the Global South met their obligations to control population growth.^16^

Robinson implies that African countries adopted population policies in exchange for foreign aid. They explained that countries with higher levels of debt were more likely to implement USAID population policies, even after accounting for other factors. They further note that the content of these policies often reflected the interests of external donors rather than local needs. Population policies from Benin, the Central African Republic, Sierra Leone, Tanzania, Togo, Ghana, Uganda, and Kenya provide examples of this dynamic.^21,23^

As has been illustrated, before its programmes were cut, USAID's focus on promoting democracy, education, and women's employment, or rescuing those in the Global South from hunger and poverty, was not the original aim of US population policy; rather, these were the means to a greater end. Significantly, the legacy of population control policies continues to fuel mistrust, which anti-rights actors exploit to challenge progressive SRHR movements. It is essential to acknowledge and confront these histories directly, instead of distorting or ignoring them.

## Implications for reproductive justice

The reproductive justice framework, which critiques the dominant conception of “choice” underpinning mainstream discussions on reproductive rights, encompasses the complete physical, mental, spiritual, political, social, and economic well-being of women and girls.^24^ It challenges the assumption that all individuals have equal access to reproductive options despite structural inequities and affirms three interrelated rights: the right to have a child, the right not to have a child, and the right to raise children in safe and healthy environments.^24,25^

USAID's significant role in expanding access to family planning information, services, and supplies in 41 countries with the greatest need aligns with the right not to have children.^26^ This is particularly important given findings by Bendavid and colleagues that reduced financial support for family planning may have led African women to substitute abortion for contraception, a significant trend in a context where most abortions are restricted and unsafe.^27^ However, the agency's history of coercive population control represents a profound reproductive injustice. By targeting impoverished women and girls, such policies pathologised their fertility and disregarded their right to have a child.

## Reckoning with the past: recommendations for accountability

As part of the process of (re)membering, there is a need for African narratives and approaches to define the continent's SRHR space. For too long, this space has been shaped by international donors, multilateral agencies, and global health institutions that positioned the 1994 ICPD as the starting point of reproductive rights discourse in Africa. We need to stop acting as though our SRHR universe, at least in communities in Africa, began in 1994, and that everything before that is insignificant. To win, the narrative war requires our honest reflection. Dismantling USAID without acknowledging its complex past will not only incur financial costs but may also threaten another vital currency in this space: legitimacy. Today, our understanding of accountability within SRHR and reproductive justice is based on the need to hold governments responsible for their obligations as outlined in various regional and global instruments. Historical accountability rarely seems to be part of that equation. This suggests a conspiracy of silence designed to obscure the truth about the institutions that are central to that space. It is hard to believe that we can speak of reproductive justice without accountability. As we mourn the loss of USAID, let us do so with honesty as part of building an equitable movement and a future grounded in truth and integrity.

In light of USAID's complex legacy and its recent structural changes, a forward-looking approach to reproductive justice is imperative. This demands the following:

1. **Formal institutional acknowledgment:** Aligning with the precedent set by organisations like MSI Reproductive Choices and Planned Parenthood, USAID or the relevant governmental entities overseeing its absorbed functions must confront its troubling history by issuing a formal apology and acknowledging its role in coercive population control policies in Africa and the rest of the Global South. This apology should explicitly recognise the profound harms caused and the violations of bodily autonomy and fundamental human rights. By openly discussing the unsavoury aspects of USAID's population control history, the spread of misinformation by anti-gender and anti-rights actors will be effectively challenged. These groups often exploit historical silences or denials of harm by organisations such as USAID to fuel mistrust and spread misinformation about the SRHR movement. Acknowledging and critically engaging with USAID's past, therefore, helps to distinguish legitimate calls for reproductive justice from manipulative narratives wrought by the anti-rights movement. These harmful narratives seek to undermine sexual and reproductive health and rights by falsely conflating historical population control coercion with contemporary initiatives.
2. **Establish comprehensive reparative mechanisms:** USAID or the entities that manage its absorbed functions must establish comprehensive reparative mechanisms. This includes, but is not limited to, directing targeted financial support to organisations in Africa working on reproductive justice from a human rights perspective. This support should specifically prioritise SRHR organisations that have consistently advanced rights-based approaches, aiming to redress historical harms and empower local agency.
3. **Prioritise Indigenous SRHR programming and domestic resource mobilisation:** Countries on the African continent must increase domestic financing for family planning and reproductive health to mitigate the funding gaps caused by USAID's withdrawal. This can be achieved through:
  - Increasing direct funding to national and regional organisations that prioritise the sexual and reproductive health needs of the African populace. Such an organisation includes Akina Mama wa Afrika, which is a Pan-African feminist organisation that strives to advance SRHR for women and girls. Other organisations include SRHR Africa Trust, which advances SRHR among adolescents and young people in Southern Africa, and the Kenya Legal and Ethical Issues Network on HIV and AIDS, which highlights SRHR as one of its core focus areas.
  - Promoting collaborative research, capacity building, and policy development between countries in the region to reduce dependence on the Global North.

## Conclusion

The dismantling of USAID marks a turning point for Africa. It is not only a loss of funding, but an opportunity for us to reflect on our reproductive self-determination, and the enduring impacts of harmful institutional legacies on our sexual and reproductive health and rights. Our demands for accountability, reparative action, and investment in Indigenous SRHR programming are not about looking backward in blame but about building forward with honesty and integrity. When institutions like USAID acknowledge the harm caused by coercive population control policies, it restores trust and protects the SRHR movement from misinformation and manipulation.

More importantly, shifting the centre of SRHR leadership to African actors, researchers, and communities will ensure that our priorities reflect the realities and aspirations of African women and girls. Increasing domestic financing and fostering regional collaboration will also help us move from dependence on foreign aid to self-sufficiency in our own health systems. This transformation reimagines reproductive justice as something rooted in truth, history, and self-determination.

## Reflexivity statement

The authors are both from Sub-Saharan Africa and are currently based in Uganda. NM (male) is Ugandan, while NK (female) is Ghanaian. Both are early-career researchers engaged in sexual and reproductive health and rights (SRHR), committed to reproductive justice across Africa. Their geographic and professional positioning offers them a critical lens on the legacies of donor-driven interventions, particularly USAID's involvement in population control policies in Africa. The authors acknowledge how their identities, lived experiences, and professional engagements shape their analysis and interpretation of these complex histories and contemporary implications for African reproductive justice. This reflexivity is crucial to situate their perspectives within the broader decolonial and rights-based approach that their work advocates.
